# Evolutionary Conservation of the Effects of Sex and Age in the Drosophila Metabolome

**DOI:** 10.1093/geroni/igaf122.2301

**Published:** 2025-12-31

**Authors:** Avani Mital, Jessica Hoffman, Ben Harrison, Daniel Raftery, Daniel Promislow

**Affiliations:** Jean Mayer USDA Human Nutrition Research Center on Aging, Boston, Massachusetts, United States; Augusta University, Augusta, Georgia, United States; University of Washington, Seattle, Washington, United States; University of Washington, Seattle, Washington, United States; Jean Mayer USDA Human Nutrition Research Center on Aging, Boston, Massachusetts, United States

## Abstract

Sex plays a crucial role in aging, influencing health trajectories, longevity, and disease susceptibility. Understanding the underlying mechanisms of these sex differences, and the extent to which they are evolutionarily conserved, remains a key challenge in aging research. Metabolites, the small molecules that mediate biochemical processes and link genetic variation with phenotypic traits, offer a powerful lens through which to explore sex-specific biology. Using a comparative framework across 11 Drosophila species, representing 50 million years of evolution, we investigate sex differences in the metabolome of young and old flies to identify patterns of age- and sex-related metabolomic changes. We find that male and female metabolomic profiles are highly correlated with each other across species, with this correlation weakening slightly with age. Despite this, numerous metabolites remain consistently sexually dimorphic across species and age groups, suggesting that sex-specific metabolic regulation is deeply evolutionarily conserved. We propose that endophenotypes like the metabolome can provide valuable insights into the mechanisms underlying sex differences in diverse traits such as age-related morbidity and mortality. Moreover, conserved and species-specific patterns can reveal why these differences have arisen and been maintained over evolutionary time.

